# Aerobic exercise modalities on gut microbiome and skeletal muscle quality in ovariectomized mice

**DOI:** 10.3389/fcimb.2025.1634934

**Published:** 2025-09-16

**Authors:** Tao Li, Yongjun Lu, Fangfang Yu, Qiuling Zhong, Yifan Meng, Yiwei Feng, Yi Hu, Xiangyang Tian, Tingting Li, Rengfei Shi

**Affiliations:** ^1^ School of Exercise and Health, Shanghai University of Sport, Shanghai, China; ^2^ School of Medical Humanities and Center for Physical Education, Anhui Medical University, Hefei, Anhui, China

**Keywords:** ovariectomy, gut microbiota, aerobic exercise, sarcopenia, mice

## Abstract

**Objective:**

This study aimed to investigate the effects of aerobic exercise on skeletal muscle quality, gut microbiota composition, and estrogen levels in ovariectomized (OVX) mice, and to elucidate the potential underlying mechanisms, thereby providing experimental evidence for exercise intervention in postmenopausal women.

**Methods:**

Adult female C57BL/6J mice were randomly assigned to four groups (n = 6 per group): Sham, OVX, Sham+ET, and OVX+ET. After 6 weeks of recovery, the exercise groups received 8 weeks of treadmill training. Muscle morphology, function, and protein metabolism pathways were assessed using histology, grip tests, and Western blotting. Aromatase and estrogen levels were evaluated by immunofluorescence and ELISA. Gut microbiota composition was analyzed via 16S rRNA sequencing and correlated with muscle function.

**Results:**

Eight weeks of aerobic exercise significantly improved skeletal muscle mass, fiber cross-sectional area, and grip strength in OVX mice, and reduced fatigue index compared to OVX controls. Immunofluorescence revealed increased aromatase expression and intramuscular E_2_ levels following exercise, with no significant difference in serum estradiol. Western blot analysis indicated activation of the Akt/mTOR/p-S6 pathway and inhibition of FOXO3-mediated protein degradation. 16S rRNA sequencing showed that exercise increased α-diversity (Shannon and Simpson indices) and altered microbial community structure, as shown by distinct clustering in PCoA plots. At the genus level, exercise modulated the relative abundance of several bacterial taxa. Spearman correlation analysis demonstrated that microbial diversity indices were positively associated with lean mass and fatigue resistance.

**Conclusion:**

Aerobic exercise significantly improves muscle mass and function in ovariectomized mice, potentially through a combined mechanism involving regulation of protein metabolism, enhancement of local estrogen synthesis, and modulation of gut microbiota composition.

## 1 Introduction

Menopause represents a critical stage in a woman’s life, during which ovarian function gradually declines, leading to a significant reduction in circulating estrogen levels (McCarthy and Raval, 2020) and triggering a series of physiological deteriorations. Among these, the decline in skeletal muscle mass and function is particularly prominent, manifesting as muscle atrophy, reduced strength, and impaired physical performance (Pellegrino et al., 2022; Lu and Tian, 2023). These changes not only significantly impair the quality of life in postmenopausal women but also markedly increase the risks of falls, fractures, and other health complications (Barron et al., 2020). Epidemiological evidence highlights the clinical significance of sarcopenia in postmenopausal women. A cross-sectional study of 340 Thai women aged 45–65 reported a prevalence of 11.8% for pre-sarcopenia and 2.7% for sarcopenia (Orprayoon et al., 2021). In another large-scale investigation of elderly postmenopausal women not receiving hormone replacement therapy, the prevalence of sarcopenia was found to be as high as 22.6% (Kenny et al., 2003). Consequently, how to effectively mitigate estrogen deficiency-induced muscle loss has garnered widespread attention and become a major research focus in exercise physiology and health sciences.

As one of the key regulators of host health, the gut microbiota interacts closely with host metabolism and immune systems and plays a critical role in the regulation of skeletal muscle mass and function (Lahiri et al., 2019; Zhang T. et al., 2022). According to the “gut–muscle axis” hypothesis, gut microbes may influence muscle metabolic homeostasis through microbial metabolites (e.g., short-chain fatty acids) (Frampton et al., 2020), inflammatory responses (Bakinowska et al., 2024), hormonal signaling pathways (Neufeld et al., 2024), and other mechanisms affecting skeletal muscle metabolic balance (Li et al., 2024). Studies have shown that aging or declining hormone levels are often accompanied by reduced microbial diversity, a decrease in beneficial bacteria such as Lactobacillus and Bacteroides, and an increase in pathogenic bacteria like Enterobacter (Picca et al., 2018). This microbial dysbiosis is frequently associated with a loss of muscle mass and decline in muscle function. Conversely, supplementing with probiotics or enhancing microbial diversity has shown promise in improving muscle function (Chen et al., 2022). Thus, targeting the gut microbiota has emerged as a potential intervention strategy. However, studies investigating how estrogen deficiency alters gut microbiota and how such changes impact skeletal muscle remain limited, particularly regarding whether these alterations can be modulated through lifestyle interventions.

Aerobic exercise, as a safe, cost-effective, and efficient non-pharmacological intervention, has been widely adopted to prevent and alleviate muscle loss in postmenopausal women (Khalafi et al., 2023; Tan et al., 2023). Research has shown that aerobic exercise can activate skeletal muscle protein synthesis pathways, such as the Akt/mTOR signaling pathway, inhibit protein degradation pathways including FOXO3a/Atrogin-1, and promote muscle protein anabolism (Zeng et al., 2020). Moreover, aerobic exercise can improve metabolic status and modulate gut microbiota diversity and composition, thereby restoring microbial homeostasis (Quiroga et al., 2020). Recent studies further suggest that regular exercise may enhance the expression of aromatase in skeletal muscle, promoting local estrogen synthesis and providing intrinsic hormonal support for muscle maintenance (Shi et al., 2019). However, most existing studies have focused on either muscle tissue or gut microbiota independently, and few have systematically investigated how aerobic exercise regulates the “gut microbiota–skeletal muscle” axis under estrogen-deficient conditions.

Therefore, this study employed an ovariectomized mouse model to comprehensively investigate the effects of aerobic exercise on skeletal muscle mass and gut microbiota composition under estrogen-deficient conditions. We evaluated changes in aromatase expression and protein synthesis/degradation signaling pathways in muscle, and further analyzed the correlations between gut microbiota diversity and skeletal muscle function. These findings are expected to provide both theoretical insights and practical guidance for maintaining muscle health in postmenopausal women, offering important implications for both basic and applied research.

## 2 Materials and methods

### 2.1 Experimental animals and grouping

Twelve-week-old healthy female C57BL/6J mice were purchased from Nanjing GemPharmatech Co., Ltd. (Nanjing, China). All experimental procedures were conducted in accordance with ethical guidelines and were approved by the Animal Care and Use Committee of Shanghai University of Sport. The mice were housed in a standard environment maintained at a temperature of 22–25 °C and relative humidity of 50%–60%, with a 12-hour light/dark cycle, and given free access to standard chow and water. Mice were randomly assigned into four groups (n = 6 per group): sham-operated control group (Sham), sham-operated plus exercise group (Sham+ET), ovariectomy group (OVX), and ovariectomy plus exercise group (OVX+ET). A group size of six was chosen based on previous studies using ovariectomized or aging mouse models that evaluated skeletal muscle and gut microbiota outcomes (Shi et al., 2019; Chen et al., 2022). Given the robust phenotype induced by OVX and the tightly controlled experimental conditions, inter-group variability was minimized. Surgical procedures were performed under isoflurane inhalation anesthesia. Bilateral ovariectomy was performed in the OVX and OVX+ET groups, while only ovarian exposure without removal was conducted in the Sham and Sham+ET groups (Hu et al., 2024). Exercise intervention began six weeks after surgery recovery.

### 2.2 Aerobic exercise intervention protocol

Mice in the exercise groups (Sham+ET and OVX+ET) first underwent a one-week treadmill acclimation program (10 m/min, 0° incline, 30 minutes per day). This was followed by an 8-week formal treadmill training regimen, conducted six days per week for 60 minutes per session. The training speed started at 12 m/min in the first week and increased by 1 m/min each subsequent week, reaching 18 m/min by the eighth week, with no incline throughout. Mice in the non-exercise groups (Sham and OVX) were allowed normal cage activity without additional interventions. No electric shocks or auditory stimuli were used during the training process (Yuan et al., 2022; Tian et al., 2025).

### 2.3 Sample collection and processing

At the end of the 8-week intervention, fresh fecal samples were collected and immediately stored at –80°C for subsequent microbiota analysis. After 12 hours of fasting, the mice were anesthetized and sacrificed. Blood samples were collected (centrifuged at 3000 rpm for 15 minutes) and stored at –80°C. Lower limb skeletal muscles including the gastrocnemius were dissected, weighed for wet mass, snap-frozen in liquid nitrogen, and stored for further analysis.

### 2.4 Body composition and skeletal muscle function assessment

Body composition was measured using an EchoMRI small-animal analyzer (EchoMRI, USA) to determine fat mass and lean mass. Muscle function was assessed via grip strength using a grip strength meter (YLS-13A, Jinan Yiyan Technology Development Co., Ltd., Jinan, China), and fatigue resistance was evaluated by inverted grid and rotarod tests using the YLS-4C apparatus (same manufacturer). The latency to fall or drop-off time was recorded (Hu et al., 2024; Tian et al., 2025).

### 2.5 Gut microbiota composition analysis

Fecal microbial DNA was extracted and the V3–V4 region of the bacterial 16S rRNA gene was sequenced using the Illumina MiSeq platform. Data were processed using the DADA2 pipeline to analyze alpha and beta diversity of the gut microbiota. Structural differences among groups were compared, and relative abundances of bacterial phyla and genera were statistically analyzed (Shin et al., 2022).

### 2.6 Serum estradiol measurement

Serum estradiol (E_2_) levels were quantified using enzyme-linked immunosorbent assay (ELISA) kits (Abcam, UK) following the manufacturer’s protocol.

### 2.7 Skeletal muscle protein expression analysis

Western blotting was performed to assess protein expression in the gastrocnemius muscle, including aromatase, key components of the protein synthesis pathway (Akt, p-Akt, mTOR, p-mTOR, S6, p-S6), and the protein degradation pathway (FOXO3, Atrogin-1). Protein band intensities were quantified using ImageJ software and normalized to GAPDH as the internal control.

### 2.8 Histological analysis of muscle tissue (H&E staining)

Gastrocnemius muscle tissues were fixed in paraffin and sectioned at a thickness of 4 μm. After deparaffinization, sections were stained with hematoxylin and eosin (H&E) to visualize myofiber nuclei and cytoplasmic structures. Microscopic images were captured, and the cross-sectional area (CSA) of muscle fibers was quantified using ImageJ software. The mean value was calculated from four randomly selected fields per sample.

### 2.9 Immunofluorescence staining

Paraffin-embedded gastrocnemius sections were deparaffinized and subjected to antigen retrieval, followed by blocking with 10% donkey serum at 37°C for 1 h. Primary antibodies against aromatase and E2 were incubated overnight at 4°C. After washing with TBST, sections were incubated with fluorescent secondary antibodies (Alexa Fluor 488/555) in the dark for 1 h. Nuclei were counterstained with DAPI. Fluorescent images were captured using a laser confocal microscope, and expression levels of aromatase and E2 were analyzed (Tian et al., 2025).

### 2.10 Statistical analysis

All statistical analyses were performed using SPSS version 25.0. Data are expressed as mean ± standard deviation (SD). One-way analysis of variance (ANOVA) was used for comparisons among groups, followed by LSD *post hoc* test for pairwise comparisons. Pearson correlation analysis was conducted to assess the relationship between gut microbiota diversity indices and skeletal muscle functional parameters. A P-value < 0.05 was considered statistically significant.

## 3 Results

### 3.1 Establishment and validation of the ovariectomized mouse model

**Figure 1:**
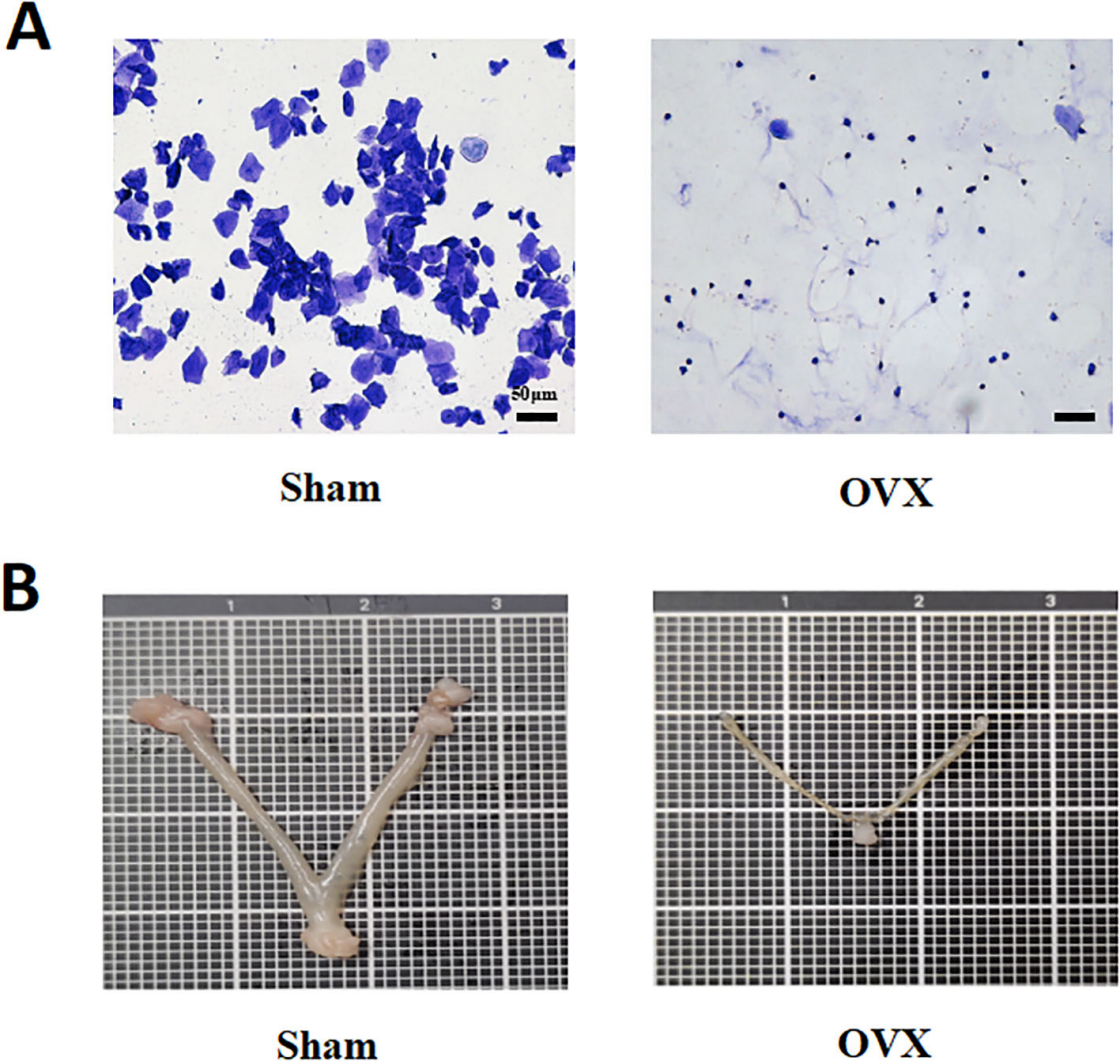
Establishment and validation of the ovariectomized mouse model. **(A)** Vaginal smear analysis shows a complete estrous cycle in the Sham group, while OVX mice exhibit persistent leukocyte dominance, indicating estrous cycle disruption and reduced estrogen levels. Scale bar = 50 μm. **(B)** Uterine morphology reveals significant uterine atrophy in OVX mice, confirming the success of ovariectomy.

To validate the successful establishment of the ovariectomized (OVX) model, the estrous cycle of mice was monitored using vaginal smear analysis, and the uterus was anatomically examined. In the Sham group, vaginal smears revealed a complete estrous cycle consisting of keratinized cells, nucleated epithelial cells, and leukocytes. In contrast, OVX mice consistently exhibited smears dominated by leukocytes, indicating a disrupted estrous cycle and significantly reduced estrogen secretion (**Figure 1A**). Additionally, the uterine size in OVX mice was markedly reduced, further confirming the success of the ovariectomy procedure (**Figure 1B**).

### 3.2 Effects of aerobic exercise on body composition and skeletal muscle function in OVX mice

**Figure 2:**
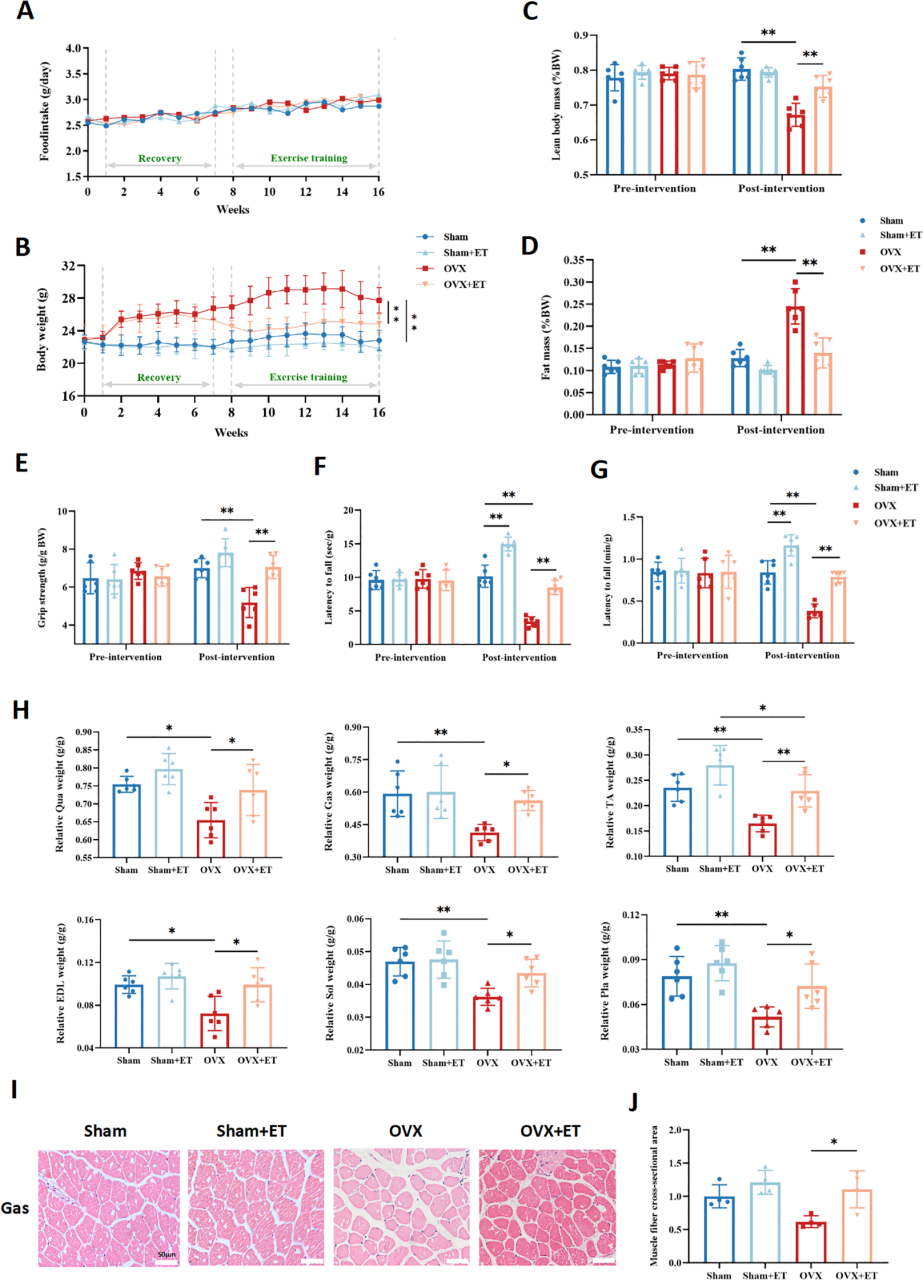
Effects of aerobic exercise on body composition and skeletal muscle function in OVX mice. **(A)** No significant differences in food intake were observed among the four groups throughout the experiment. **(B)** OVX mice showed a significant increase in body weight during the intervention period, which was markedly suppressed by aerobic exercise in the OVX+ET group. **(C)** After exercise intervention, the percentage of lean body mass was significantly higher in the OVX+ET group compared to the OVX group. **(D)** The fat mass percentage was significantly reduced in the OVX+ET group, indicating improved fat accumulation. **(E)** Grip strength was significantly enhanced in OVX+ET mice, reflecting improved muscle force. **(F)** In the inverted grid test, hanging time was significantly prolonged in the OVX+ET group, suggesting enhanced fatigue resistance. **(G)** In the rotarod test, OVX+ET mice exhibited longer latency to fall, indicating improved endurance capacity. **(H)** Relative muscle weight of lower limb muscles (quadriceps, gastrocnemius, tibialis anterior, extensor digitorum longus, soleus, and plantaris) was significantly reduced in the OVX group but markedly restored after exercise. **(I)** H&E staining sections of gastrocnemius muscle showed a reduction in muscle fiber cross-sectional area in OVX mice, which was improved after exercise intervention. **(J)** Quantification of muscle fiber cross-sectional area confirmed the histological findings. Data are presented as mean ± SD, n = 6 per group. *P < 0.05, **P < 0.01.

An 8-week aerobic exercise intervention had no significant effect on food intake among the groups (**Figure 2A**). Compared with the OVX group, the OVX+ET group exhibited a significantly attenuated trend in body weight gain (**Figure 2B**, P < 0.01). Following the intervention, lean mass percentage in the OVX+ET group was significantly higher than in the OVX group (**Figure 2C**, *P*<0.01), while fat mass percentage was markedly reduced (**Figure 2D**, P < 0.01). Functional assessments of skeletal muscle showed that aerobic exercise significantly improved grip strength in OVX mice (**Figure 2E**, P < 0.01) and enhanced fatigue resistance, as indicated by prolonged latency to fall in the inverted grid and rotarod tests (**Figures 2F, G**, P < 0.01). Moreover, the relative mass of lower limb muscles—including the quadriceps, gastrocnemius, tibialis anterior, extensor digitorum longus, soleus, and plantaris—was significantly increased in the OVX+ET group compared with the OVX group (**Figure 2H**, *P < 0.05 or **P < 0.01). H&E staining of the gastrocnemius further revealed that aerobic exercise markedly improved muscle fiber morphology and cross-sectional area in OVX+ET mice compared with OVX controls (**Figures 2I, J**, *P < 0.05).

### 3.3 Effects of aerobic exercise on gut microbiota composition in OVX mice

**Figure 3:**
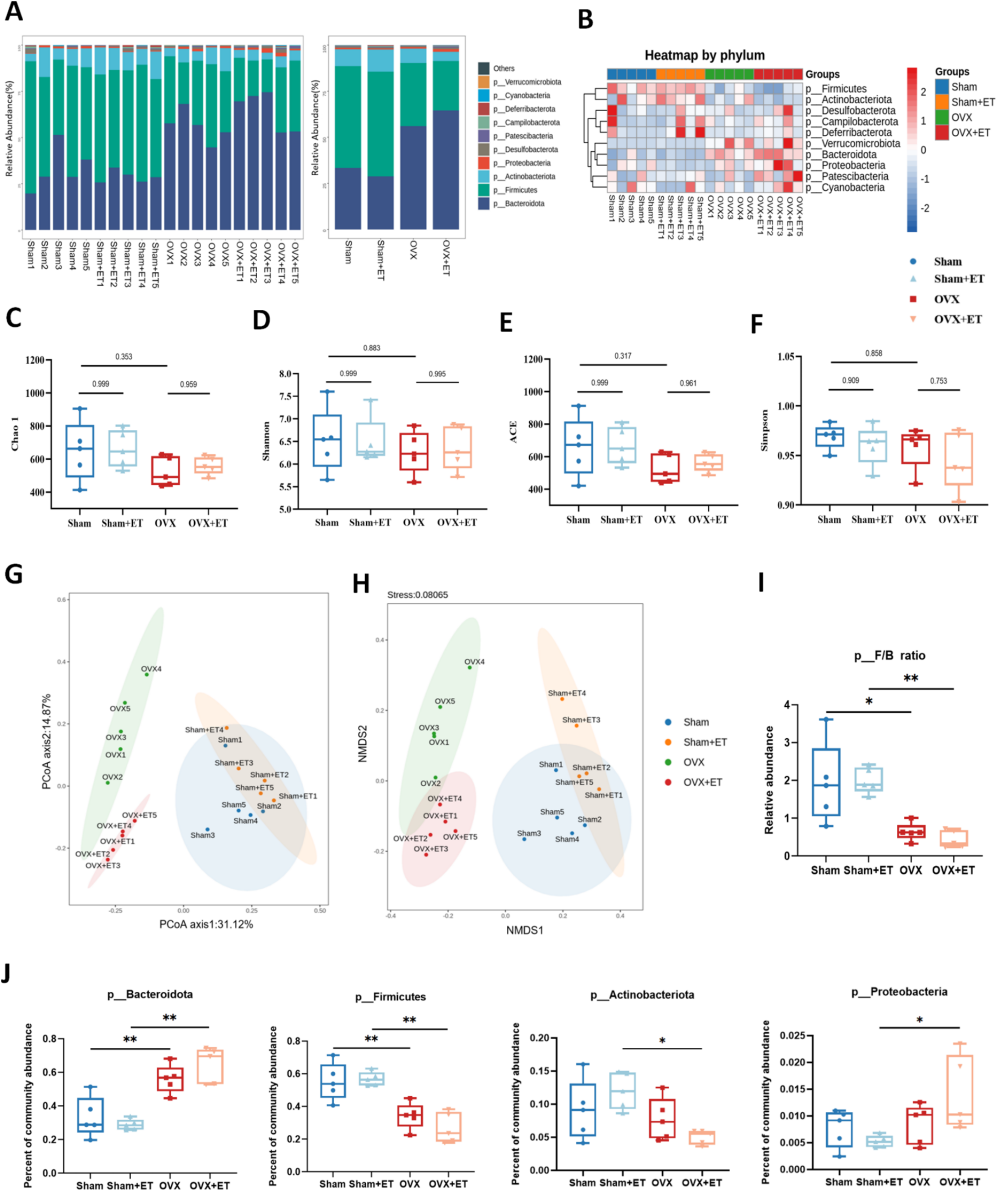
Effects of aerobic exercise on gut microbiota composition in OVX mice. **(A)** Bar plots of relative abundance at the phylum level show distinct differences in microbial composition among the four groups. **(B)** Heatmap of phylum-level taxa indicates marked compositional differences, with clear separation between OVX and Sham groups. **(C–E)** α-diversity indices including Chao1, Shannon, and ACE were significantly decreased in the OVX group compared to Sham, while OVX+ET showed a recovery trend without statistical significance. **(F)** No significant differences in Simpson index were observed among the groups. **(G, H)** PCoA and NMDS analyses demonstrated distinct clustering of OVX and Sham groups; the OVX+ET group shifted toward the Sham group but did not fully overlap. **(I)** The Firmicutes/Bacteroidota (F/B) ratio was significantly reduced in the OVX group and was not restored by exercise. **(J)** The relative abundance of Bacteroidota increased and Firmicutes decreased significantly in OVX mice; aerobic exercise did not fully reverse these changes. Data are presented as mean ± SD, n = 5 per group. *P < 0.05, **P < 0.01.

Aerobic exercise intervention significantly modulated the gut microbiota composition in OVX mice (**Figures 3A, B**). In terms of α-diversity, no statistically significant differences were observed among the four groups for the Chao1, ACE, Shannon, and Simpson indices (*P* > 0.05; **Figures 3C–F**); however, the OVX+ET group exhibited upward trends in all indices compared to the OVX group. β-diversity analysis further confirmed this trend, as both principal coordinate analysis (PCoA) and non-metric multidimensional scaling (NMDS) demonstrated a distinct separation between the OVX and Sham groups, while the OVX+ET group showed a clustering pattern more closely aligned with the Sham group (**Figures 3G, H**). The Firmicutes/Bacteroidota (F/B) ratio was significantly reduced in the OVX group (**Figure 3I**, P < 0.01). At the phylum level, compared to the Sham group, the OVX group showed a significant increase in Bacteroidota and a decrease in Firmicutes. Compared to the OVX group, the OVX+ET group showed a trend toward increased relative abundance of Bacteroidota and Proteobacteria, along with decreased Firmicutes and Actinobacteriota (**Figure 3J**).

### 3.4 Effects of aerobic exercise on aromatase and estradiol expression in skeletal muscle of OVX mice

**Figure 4:**
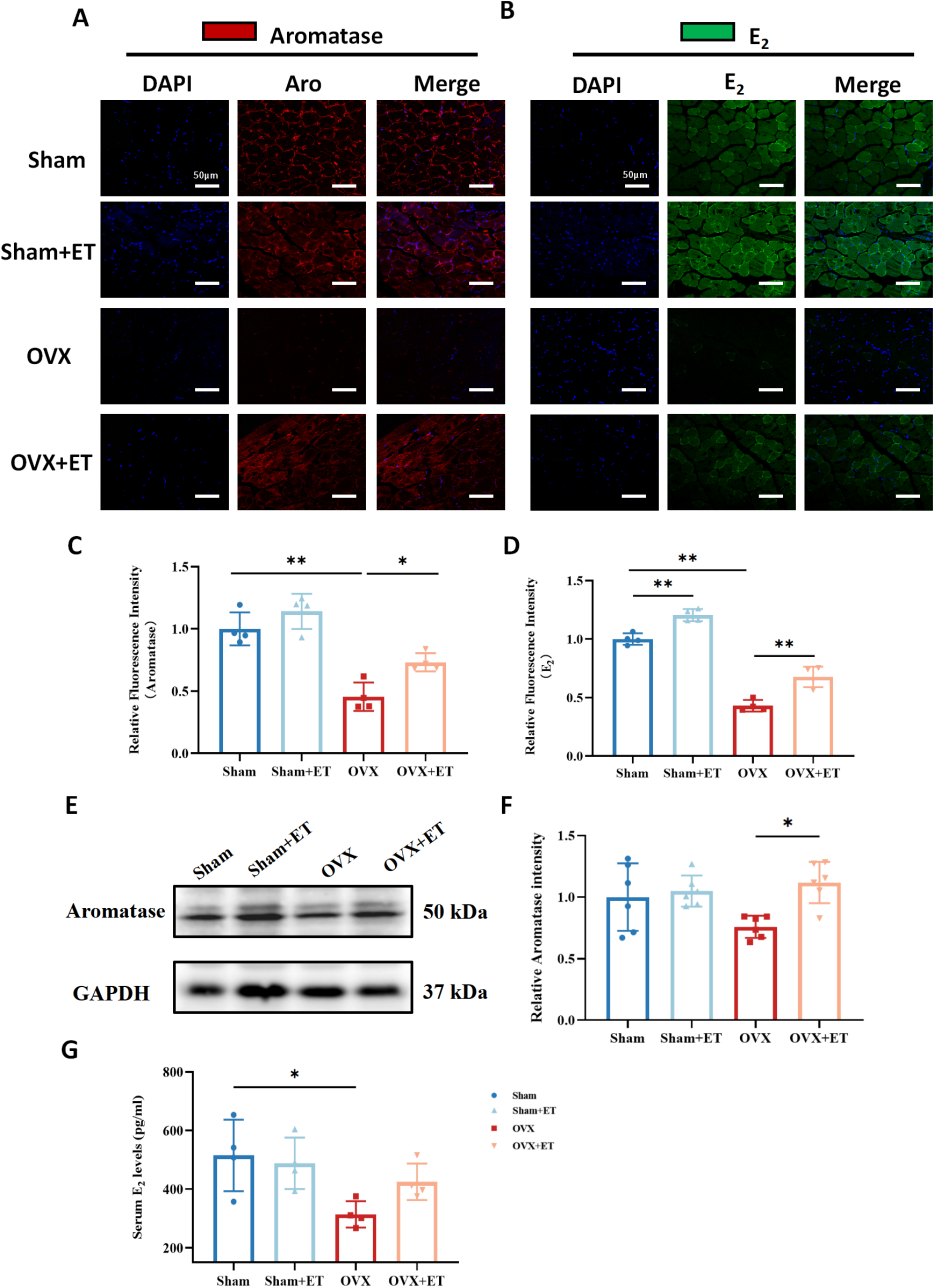
Effects of aerobic exercise on aromatase and estradiol (E_2_) expression in the skeletal muscle of OVX mice. **(A)** Immunofluorescence staining of aromatase (red) in the gastrocnemius muscle. OVX mice showed markedly reduced aromatase expression, which was partially restored after aerobic exercise (OVX+ET). **(B)** Immunofluorescence staining of estradiol (E_2_, green) revealed decreased E_2_ levels in the OVX group, with partial recovery following exercise intervention. Nuclei were counterstained with DAPI (blue). Scale bar: 50 μm. **(C, D)** Quantification of fluorescence intensity showed significantly lower aromatase and E_2_ levels in the OVX group, with significant improvement after exercise. **(E)** Western blot analysis of aromatase protein expression in gastrocnemius muscle; GAPDH was used as the internal control. **(F)** Densitometric analysis showed that aromatase protein levels were significantly higher in the OVX+ET group compared to the OVX group. **(G)** Serum E_2_ levels were significantly decreased in the OVX group and showed partial recovery after exercise. Data are presented as mean ± SD, n = 4/6 per group. *P < 0.05, **P < 0.01.

Immunofluorescence staining showed that aromatase expression in the gastrocnemius muscle was significantly reduced in the OVX group compared to the Sham group, whereas aerobic exercise markedly increased aromatase levels in the OVX+ET group compared to the OVX group (**Figures 4A, C**; *P* < 0.05). Similarly, fluorescence intensity of estradiol (E_2_) in muscle tissue exhibited a comparable pattern, with a significant reduction in the OVX group and a marked restoration in the OVX+ET group (**Figures 4B, D**; *P* < 0.01). Western blot analysis further confirmed this trend, showing decreased aromatase protein expression in the OVX group and significant upregulation following exercise intervention (**Figures 4E, F**; *P* < 0.05). In addition, serum E_2_ levels were significantly reduced in OVX mice and partially restored after aerobic exercise (**Figure 4G**; *P* < 0.05).

### 3.5 Effects of aerobic exercise on protein synthesis and degradation pathways in skeletal muscle of OVX mice

**Figure 5:**
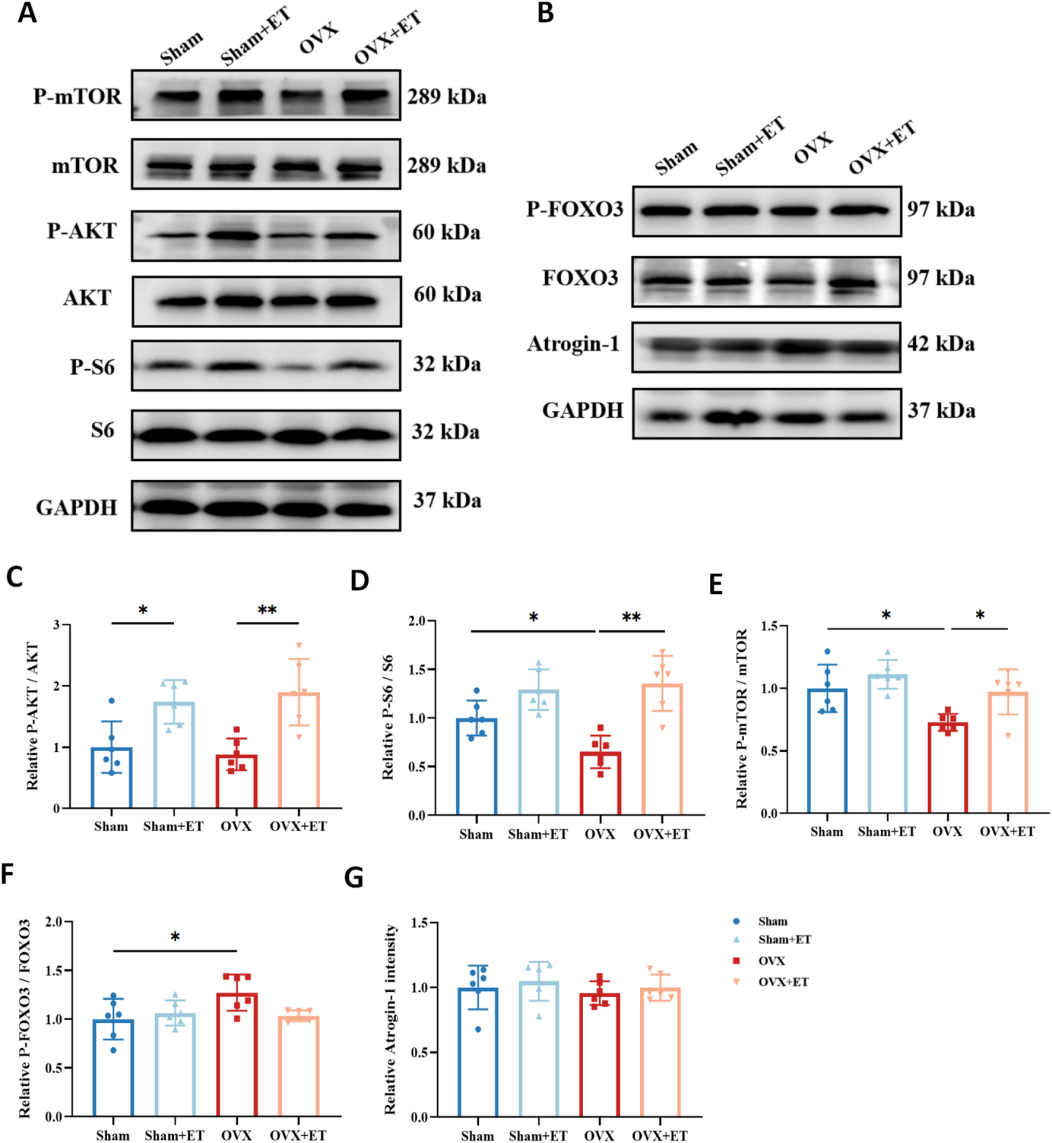
Effects of aerobic exercise on protein synthesis and degradation pathways in skeletal muscle of OVX mice. **(A)** Representative Western blot images showing the expression of proteins involved in the anabolic pathway, including Akt, phosphorylated Akt (p-Akt), mTOR, phosphorylated mTOR (p-mTOR), S6, and phosphorylated S6 (p-S6), with GAPDH used as the loading control. **(B)** Representative Western blot images showing the expression of proteins associated with the catabolic pathway, including FOXO3, phosphorylated FOXO3 (p-FOXO3), and Atrogin-1, with GAPDH as the internal control. **(C–E)** Quantification of the anabolic pathway proteins indicates that OVX significantly reduced the phosphorylation levels of Akt, mTOR, and S6, while aerobic exercise (OVX+ET) restored these levels, suggesting activation of protein synthesis signaling. **(F)** The p-FOXO3/FOXO3 ratio was significantly increased in the OVX group and was attenuated following aerobic exercise, indicating suppressed protein degradation activity. **(G)** Atrogin-1 expression did not show significant differences among the groups. Data are presented as mean ± SD, n = 6. *P < 0.05, **P < 0.01.

Western blot analysis revealed that the expression levels of phosphorylated Akt (p-Akt/Akt), phosphorylated S6 (p-S6/S6), and phosphorylated mTOR (p-mTOR/mTOR), which are key components of the protein synthesis pathway, were significantly reduced in the OVX group compared with the Sham group (*P* < 0.05). In contrast, aerobic exercise significantly upregulated the phosphorylation levels of these proteins in the OVX+ET group, indicating effective activation of the Akt/mTOR/S6 protein synthesis signaling pathway by exercise (**Figures 5A, C–E**; *P* < 0.05 or *P* < 0.01). Regarding the protein degradation pathway, the level of phosphorylated FOXO3 (p-FOXO3/FOXO3) was significantly elevated in the OVX group, suggesting enhanced proteolytic activity, whereas aerobic exercise reduced this expression to near-normal levels (**Figures 5B, F**; *P* < 0.05). However, no significant differences in Atrogin-1 expression were observed among the groups (**Figures 5B, G**).

### 3.6 Correlations between gut microbiota α-diversity indices and skeletal muscle function or body composition in mice

**Figure 6:**
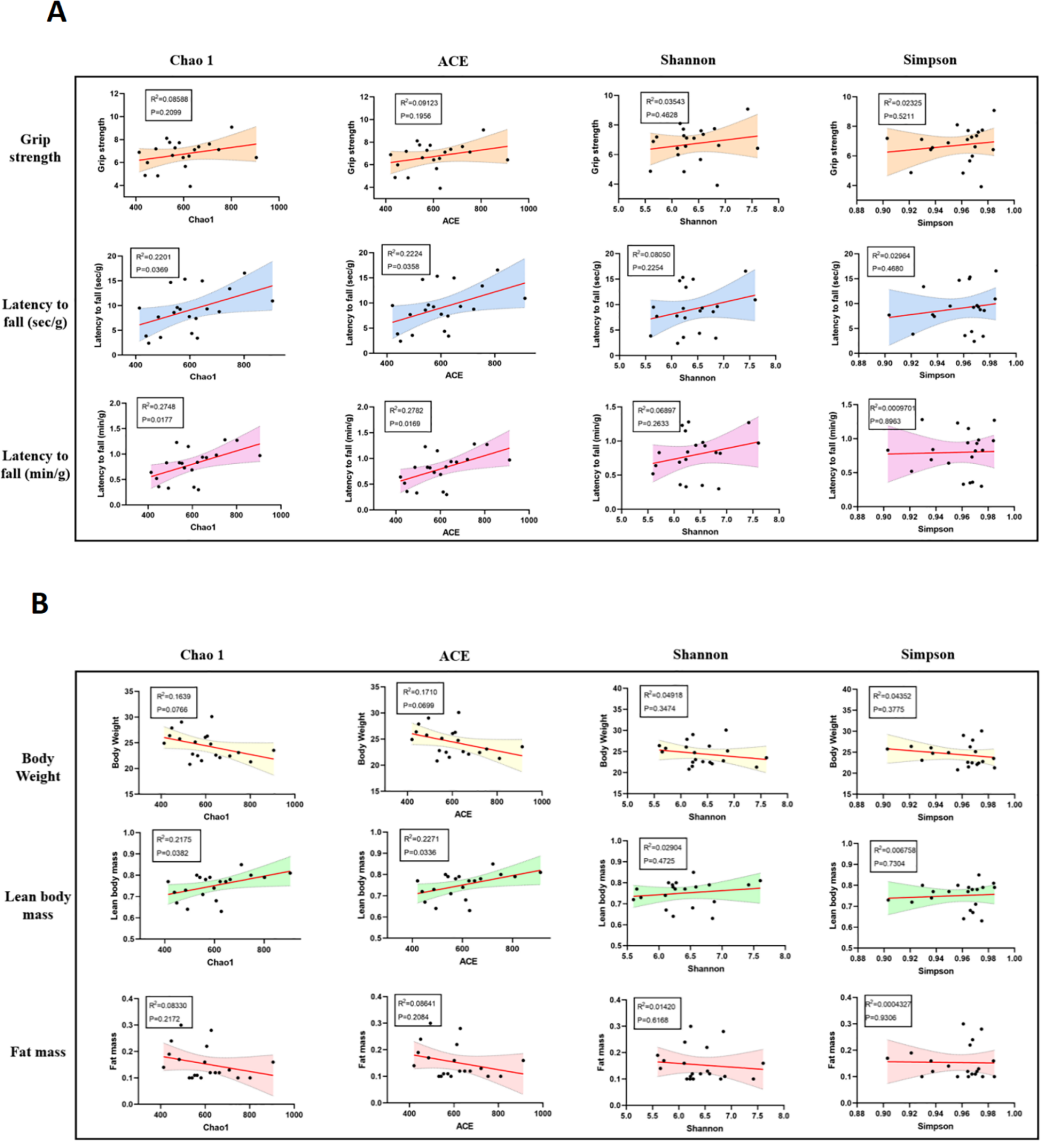
Correlations between gut microbiota α-diversity indices and skeletal muscle function or body composition in mice. **(A)** Pearson correlation analysis between α-diversity indices (Chao1, ACE, Shannon, and Simpson) and skeletal muscle function indicators. Chao1 and ACE indices showed significant positive correlations with latency to fall in the inverted grid test and rotarod test, suggesting that higher microbial diversity is associated with better muscle endurance. **(B)** Correlation analysis between gut microbiota diversity and body composition parameters. Chao1 and ACE indices were positively correlated with lean body mass, while no significant correlations were observed between diversity indices and fat mass or body weight. Shaded areas represent 95% confidence intervals of the regression lines. *P* < 0.05 was considered statistically significant.

Further correlation analysis demonstrated a certain degree of association between gut microbiota α-diversity and skeletal muscle function. Both the Chao1 and ACE diversity indices were significantly and positively correlated with fatigue resistance, as measured by the inverted grid and rotarod tests (**Figure 6A**), suggesting that higher microbial diversity is associated with better muscular endurance. Regarding body composition, the Chao1 and ACE indices also showed a significant positive correlation with lean mass, whereas no statistically significant correlations were found with fat mass or body weight (**Figure 6B**).

## 4 Discussion

This study, based on an estrogen-deficient mouse model, systematically evaluated the regulatory effects of aerobic exercise on skeletal muscle function and gut microbiota. The results demonstrated that 8 weeks of aerobic exercise significantly improved muscle mass and function in ovariectomized (OVX) mice, restored gut microbiota composition, upregulated aromatase expression and local estrogen levels in skeletal muscle, activated protein synthesis pathways, and suppressed protein degradation. Moreover, microbial diversity was significantly correlated with muscle function, suggesting that the “gut microbiota–skeletal muscle” axis may represent a critical regulatory pathway.

Aerobic exercise, as a non-pharmacological intervention, has been widely demonstrated to improve gut microbiota composition (Resende et al., 2021). Studies have shown that regular physical activity can promote the colonization of probiotics (such as Lactobacillus and Bacteroides) and suppress the overgrowth of pathogenic bacteria by enhancing intestinal peristalsis, improving intestinal barrier function, and reducing systemic inflammation, thereby increasing microbial diversity and restoring metabolic homeostasis (Aragón-Vela et al., 2021; Dmytriv et al., 2024). In the present study, 8 weeks of aerobic exercise significantly altered the gut microbial composition of OVX mice, restoring the relative abundance of beneficial phyla such as Bacteroidota, suggesting that exercise may regulate skeletal muscle function through the “gut–muscle axis”. Notably, aerobic exercise markedly increased gut microbial diversity in the OVX group but not in sham-operated mice, suggesting a differential response based on estrogen status. Estrogen deficiency disrupts gut barrier integrity and microbial homeostasis, making OVX mice more responsive to exercise-induced microbial restoration. In contrast, the sham group maintains a relatively stable microbiota, with exercise exerting only minor structural adjustments. These findings imply that the divergent microbial changes result from distinct host–microbiota interactions under different hormonal conditions. In addition, microbial-derived short-chain fatty acids (e.g., acetate and butyrate) have been reported to activate the AMPK pathway and inhibit histone deacetylases, thereby promoting protein synthesis and mitochondrial biogenesis in skeletal muscle (Hu et al., 2018; Frampton et al., 2020). This may represent an indirect mechanism by which exercise improves muscle function. Furthermore, our findings revealed that exercise markedly upregulated aromatase expression in skeletal muscle, accompanied by an increase in local E_2_ levels. Previous studies have indicated that aromatase expression is regulated by transcription factors such as AMPK and PGC-1α, which can be activated by exercise (Zhang et al., 2024). Exercise-induced upregulation of aromatase may enhance local estrogen synthesis in muscle, facilitating muscle fiber repair and functional recovery (Pellegrino et al., 2022; Seibert et al., 2024), suggesting that skeletal muscle may act as a target-like tissue for exercise-induced local estrogen production. In support of this, we observed increased aromatase expression and estrogen levels in skeletal muscle following exercise, suggesting a potential for local synthesis (Shi et al., 2019). However, due to the lipophilic nature of steroid hormones and the rich vascularization of muscle tissue, passive uptake from peripheral circulation cannot be excluded (Miller and Auchus, 2011). Moreover, the current analytical methods are insufficient to determine the exact origin of intramuscular estrogen. Future studies could consider muscle-specific knockout models, local inhibition strategies, or metabolic tracing techniques to further clarify the source.

In recent years, growing evidence has suggested that the gut microbiota can influence skeletal muscle mass by regulating energy metabolism, mitochondrial biogenesis, and inflammatory responses through its metabolites, such as short-chain fatty acids and secondary bile acids (Imdad et al., 2022; Lian et al., 2022). This relationship is particularly relevant under conditions of estrogen deficiency, where a decline in microbial diversity is closely associated with sarcopenia. Guo et al. demonstrated in an ovariectomized mouse model that dysbiosis of the gut microbiota is strongly linked to enhanced muscle protein degradation, while probiotic supplementation can significantly improve muscle function (Guo et al., 2023). More importantly, we observed that aerobic exercise markedly upregulated aromatase expression in skeletal muscle, along with a recovery in local E_2_ levels, suggesting that skeletal muscle may serve as a peripheral estrogen-producing organ in response to exercise. The expression of aromatase in muscle tissue can be activated by exercise, potentially through the ERK/CREB signaling pathway, thereby enhancing local estrogen synthesis (Mori et al., 2019). Emerging evidence indicates that endurance training exerts bidirectional regulatory effects on both skeletal muscle and the gut microbiota. On one hand, aerobic exercise can enhance local estrogen signaling in skeletal muscle by upregulating intramuscular aromatase expression and activating the PI3K/Akt/mTOR pathway, thereby promoting muscle regeneration in estrogen-deficient models (Saponaro et al., 2024). On the other hand, endurance exercise is known to increase gut microbial diversity and alter community structure, influencing host endocrine and metabolic responses. For example, Zhang et al. demonstrated that sustained high-intensity aerobic training improved muscle performance via a gut microbiota–testosterone axis in mice, confirmed through microbiota transplantation experiments (Zhang L. et al., 2022). Similarly, Marsh et al. reported that aerobic exercise enhanced metabolic and muscular health in ovariectomized mice, linked to modulation of both systemic hormone levels and gut microbial composition (Marsh et al., 2023). These findings support the existence of an integrated muscle–gut–endocrine axis through which physical training mediates coordinated adaptations in hormonal balance and skeletal muscle function.

Although this study preliminarily revealed the multifaceted beneficial effects of aerobic exercise on skeletal muscle and gut microbiota in an estrogen-deficient mouse model, several limitations should be acknowledged. First, the current findings are based on correlation analyses, and causal relationships were not verified using strategies such as fecal microbiota transplantation, antibiotic-mediated microbiota depletion, or single-strain probiotic supplementation (Rosshart et al., 2019). Second, the precise source of estrogen synthesis remains unclear, and whether increased aromatase expression directly contributes to elevated local E_2_ production requires further investigation. Third, as this study was conducted solely in a mouse model, the translational relevance to humans—particularly postmenopausal women—needs to be validated through clinical samples and intervention studies. In the future, integrative multi-omics approaches, including metabolomics and transcriptomics, may help to elucidate the specific molecular and metabolic mechanisms underlying the interplay among exercise, gut microbiota, hormones, and skeletal muscle.

## 5 Conclusion

This study demonstrates that ovariectomy-induced estrogen deficiency leads to reduced skeletal muscle mass and function in mice, accompanied by decreased gut microbiota diversity. An 8-week aerobic exercise intervention effectively ameliorated these abnormalities by enhancing muscle quality and fatigue resistance, modulating protein metabolism–related signaling pathways, and reshaping gut microbiota composition. These findings suggest that exercise may exert synergistic effects through the “gut microbiota–skeletal muscle” axis, offering a potential intervention strategy for the prevention and management of menopause-associated sarcopenia.

## Data Availability

The original contributions presented in the study are included in the article/supplementary material. Further inquiries can be directed to the corresponding author.
